# Belowground carbon transfer across mycorrhizal networks among trees: Facts, not fantasy

**DOI:** 10.12688/openreseurope.16594.1

**Published:** 2023-10-03

**Authors:** Tamir Klein, Ido Rog, Stav Livne-Luzon, Marcel G.A. van der Heijden, Christian Körner

**Affiliations:** 1 Weizmann Institute of Science, Rehovot, Center District, Israel; 2 agroscope, Zuerich, Switzerland; 3 university of basel, Basel, Switzerland

**Keywords:** common mycorrhizal network, belowground carbon transfer, root carbon uptake, mycorrhizal exchange, isotopic carbon labeling, dual mycorrhization

## Abstract

The mycorrhizal symbiosis between fungi and plants is among the oldest, ubiquitous and most important interactions in terrestrial life on Earth. Carbon (C) transfer across a common mycorrhizal network (CMN) was demonstrated over half a century ago in the lab ( Reid & Woods, 1969), and later in the field ( Simard *et al*., 1997a). Recent years have seen ample progress in this research direction, including evidence for ecological significance of carbon transfer ( Klein *et al*., 2016). Furthermore, specific cases where the architecture of mycorrhizal networks have been mapped ( Beiler *et al*., 2015) and CMN-C transfer from mature trees to seedlings has been demonstrated ( Orrego, 2018) have suggested that trees in forests are more connected than once thought ( Simard, 2021). In a recent *Perspective*, Karst *et al*. (2023) offered a valuable critical review warning of over-interpretation and positive citation bias in CMN research. It concluded that while there is evidence for C movement among plants, the importance of CMNs remains unclear, as noted by others too ( Henriksson *et al*., 2023). Here we argue that while some of these claims are justified, factual evidence about belowground C transfer across CMNs is solid and accumulating.

## Plain language summary

Mycorrhizas are fungal associations between plant roots and beneficial fungi (mushrooms). In forests, some of these belowground associations can include more than one tree, creating a common mycorrhizal network (CMN). It has been shown in multiple studies that CMNs can serve as transport pathways of carbon among different trees. Recently, Karst *et al*. (2023) offered a valuable critical review questioning the importance of CMNs, as noted by others too ( Henriksson *et al*., 2023). Here we argue that factual evidence about belowground C transfer across CMNs is solid and accumulating and discuss current questions in CMN research.

Recently, Cahanovitc *et al*. (2022) showed unequivocally, using DNA-stable isotope probing, ^13^C in the DNA of specific mycorrhizal species colonizing roots of both donor and recipient saplings, growing in forest soil under natural conditions. In addition, the label was found not on roots only, but also in stems, as previously seen in mature trees in the forest ( Klein *et al*., 2016). Remaining questions are (1) Why is CMN-C transfer so elusive? (2) How important are alternative transfer pathways? (3) What is the significance for trees? (4) How can we explain the counterintuitive C transfer from fungus to the recipient tree? And finally, (5) What is the benefit to the fungus? The next few paragraphs offer answers to these key questions.

(1) Why is CMN-C transfer so elusive? Multiple labeling experiments detect CMN-C transfer, while others do not ( Karst *et al*., 2023). Often, this is because labeling intensity is too low to detect an otherwise small C flow due to high labeling material costs. Regardless, chances are meager to collect the specific root with specific mycorrhizal fungi at the exact time of C transfer due to the spatial and dynamic complexity of the CMN ( Read *et al*., 1985). The mycorrhizal community of mature trees differs on every root, even among root tips on the same root branchlet ( Rog *et al*., 2020; Rog *et al*., 2022). Furthermore, trees allocate different amounts of C to varying roots according to soil niche, microbial community, and other root trait parameters. Trees of various species occupy different soil niches; mycorrhizal species are also located in different depths ( Toju *et al*., 2016). The ecological significance of C transfer among different tree species can be masked by the complexity of host tree roots and CMNs.

(2) How important are alternative transfer pathways? These include respiration, exudation, turnover, mass flow, assimilation, and redistribution by soil biota ( Henriksson *et al*., 2023). These pathways probably can never be completely ruled out. However, C flow through fungal mycelium is much more efficient than through bulk soil because it bypasses soil microbial assimilation and transformation. Diffusional mass flow in unsaturated soil is in the magnitude of m month ^-1^, and its temporal dynamics rarely match those of the observed label transfer ( Avital *et al*., 2022). In addition, exudates rarely travel more than a few mm in soil without active transport ( Kuzyakov *et al*., 2003). It is obviously possible that one fungus’ hyphae exudate C compounds that are consequently acquired by neighboring fungal hyphae (not belonging to the same mycelium; Karst *et al*., 2023). Still, a forest study showed lack of label transfer to plants hosting other mycorrhizal types and lack of label transfer to saprotrophic fungi ( Klein *et al*., 2016). Simard *et al*. (1997a) also found a fraction of C label transferred to tree seedlings not involved in a CMN compared to those that were. These studies support the notion that C is moving through hyphal networks from one plant to another.

(3) What is the significance for trees? A traditional view often measures benefit to trees based on growth enhancement, such as stem height, diameter, or biomass. However, it should be noted that C transfer is typically small compared to autotrophic C assimilation, making it less likely to have a direct impact on the recipient’s growth. Hence, the significance of the CMN-C transfer is probably more nuanced, e.g., in providing C for osmoregulation ( Sapes *et al*., 2021) or defense metabolite transfer ( Song *et al*., 2015). It is also important to mention that many plants (including trees) are nutrient-limited and not C-limited ( Körner, 2015; Kiers & van der Heijden, 2006). There are probably no strong evolutionary selection pressures to prevent C loss if C is a luxury good through most of the lifespan of a tree and if delivering this C to the CMN is also linked to benefits (e.g. nutrients provided by the CMN). Likewise, trees that may benefit from it, are limited in C source (e.g., due to deep shade; Simard *et al*., 1997b) or are either disproportionally short in C sources compared to adjacent bigger and older trees ( Simard *et al*., 1997b).

(4) How can we explain the counterintuitive C transfer from fungus to the recipient tree? Is it physiologically feasible? Levels of hexose are consistently higher in the host roots compared to the fungus, making it difficult for hexose to move against this gradient ( Henriksson *et al*., 2023). This holds true in most situations. However, there is an exception when the recipient tree is subjected to heavy shading. In such cases, the roots of the recipient tree may experience C depletion, as suggested by Sapes *et al*. (2021), thereby reversing the hexose "gradient" from fungi to roots. Moreover, C has been shown to transfer to host trees along with N, most likely in amino acids ( Teste *et al*., 2009).

(5) What is the benefit to the fungus? C flow from the donor tree is clear, given that the fungus is inherently heterotrophic, thus C-supply dependent. C flow also exists from fungus to plant: myco-heterotrophic plants that lack chlorophyll obtain C from other plants by parasiting on CMNs throughout their lifespan ( Leake, 2005). Moreover, approximately 25,000 orchid species live in symbiosis with mycorrhizal fungi ( van der Heijden *et al*., 2008). Young orchid seeds are extremely small (0.3–14 µg), lack chlorophyll and it is thought that the germinating seeds (protocorms) of almost all orchids obtain C and nutrients from their mycorrhizal symbionts, sometimes for years, before a green and autotrophic plant emerge ( Cameron *et al*., 2008). These are clear examples that C transfer from CMN to plants does occur. Yet what is the adaptive advantage of C flow from fungus to an autotrophic recipient tree? Mycorrhizal symbiosis is traditionally viewed as a belowground, cross-kingdom, exchange of carbohydrates (plant→fungus) for nutrients (fungus→plant). However, contemporary research offers a more complex view, whereby fatty acids and lipids are transferred between mycorrhizal fungi and plant hosts ( Jiang *et al*., 2017). In addition, C transfer in the form of amino acids ( Simard *et al*., 2012) inescapably involves N transfer, going both ways. Thus, a fungus→plant C movement is not unlikely when C is tied to nutrients (e.g. amino acids) or when trees or plants acquire C from degenerating hyphae in the Hartig Net (as in AMF arbuscules).

The above point (5) aligns with the notion that competition for light, water, and nutrients is still a major interaction in forests ( Henriksson *et al*., 2023; Klein *et al*., 2016). Specific mycorrhizal fungi transfer C among trees, including from canopy trees to seedlings, or from sunlit saplings to shaded saplings of the same, or different (non-kin), species. This facilitative behavior has been demonstrated by Teste *et al*. (2009) and Bingham & Simard (2012) in temperate forests but requires further testing in different biomes. C transfer takes place between different, unrelated tree species sharing mycorrhizal species ( Avital *et al*., 2022; Rog *et al*., 2020). This includes C transfer between an EM-host to an AM-host with dual mycorrhization status ( *Cupressus sempervirens*; Avital *et al*., 2022). Interestingly, some of the mycorrhizal species involved in C transfer among temperate trees (e.g. *Russula chloroides*; Rog *et al*., 2020) and among Mediterranean trees (e.g. *Tomentella ellisii*; Cahanovitc *et al*., 2022) were also identified in myco-heterotrophic plants ( Girlanda *et al*., 2006; Julou *et al*., 2005).

Finally, an evolutionary advantage should exist for fungi to maintain diversity of tree hosts and hence C sources ( Tedersoo *et al*., 2020). For example, in a mixed Mediterranean forest, tree species diverge in their phenologies and functions ( Rog *et al*., 2021), and multi-host EMF and AMF form the vast majority of mycorrhizal species ( Rog *et al*., 2022). Indeed, mycorrhizal fungi are not merely conduits for tree-tree resource sharing, but rather complex organisms having their own strategies ( Henriksson *et al*., 2023). Without a mechanism for tree-directed C transfer across CMNs, it is most probably driven by the fungi, rather than by the trees. There is a need for more experimental studies to visualize that a single mycorrhizal mycelium interconnects different trees and to assess when and how much C is moving from one tree to another. However, there is sufficient evidence that trees in forests are connected by a CMN and transmitting C among themselves, and this can lead to new management practices that tend to the whole forest rather than individual trees, thus improving the ability of forests to cope with stress. The next few years might shed new light on how CMNs and C transfer may affect forest resilience, as the field is rapidly evolving.

## Ethics and consent

Ethical approval and consent were not required.

## Data Availability

No data are associated with this article.
