# Supplementary material for: Belowground carbon transfer across mycorrhizal networks among trees: Facts, not fantasy
Source: Open Res Eur. 2023 Oct 3;3:168. [Version 1] doi: 10.12688/openreseurope.16594.1 (PMC10751480; doi:10.12688/openreseurope.16594.1)
Supplement: Peer review history

# Reviewer report: Reviewer response for version 1

**DOI:** 10.21956/openreseurope.17913.r35982

**Published:** 2023-12-26

**Authors:** Marc-Andre Selosse

**Affiliations:** 1 Institut Universitaire de France, Paris, France

A very good, well-written and timely viewpoint, with many interesting ideas and appropriated references, bravo. It deserves urgent publication but I have a couple of comments below on missing issues. As COI, I have to cite some of my own work on mycoheterotrophic and mixotrophic plants (see my comments on this issue below*). Waiving anonymity, Marc-André Selosse

 On question 1, one may mention the temporal issue (the C flux may happen, or not, or be reversed depending on time), as nicely illustrated by Lerat S, Gauci R, Catford JG, Vierheilig H, Piché Y, Lapointe L. 2002. 14C transfer between the spring ephemeral *Erythronium americanum* and sugar maple saplings via arbuscular mycorrhizal fungi in natural stands. *Oecologia* 132: 181–187. In this paper, the plant is an understory one but the author may not forget to mention the understory which may have strong need and may be a sink for tree C.

 On question 2, C flow through fungal mycelium is also much more efficient than through bulk soil because it is directional, and thus avoids the dilution that happens in any isotropic mechanism.

 On question 3, one may mention unequal reward that may be even more relevant: different plants associated to a same fungus they support may not (i) give same C amount and (ii) get the same amount of minerals, so that one plant may indirectly subsidize the mineral acquisition of another. It is an economy of C for the ‘winning plant’ which too overlooked while it is C-relevant! See (although they do not use trees) Florian Walder, Helge Niemann, Mathimaran Natarajan, Moritz F. Lehmann, Thomas Boller, Andres Wiemken, Mycorrhizal Networks: Common Goods of Plants Shared under Unequal Terms of Trade, *Plant Physiology*, Vol 159, 2, June 2012, Pages 789–797, https://doi.org/10.1104/pp.112.195727

 On question 4 (see my comments on this issue below*), one may mention mycoheterotrophic and mixotrophic plants, where evidences point toward amino-acids as C source (Lallemand et al., 2019. In situ transcriptomic and metabolomic approach to the transition to the loss of photosynthesis in plants exploiting fungi. The Plant Journal 98: 826-841; Fochi et al., 2017. Fungal and plant gene expression in the Tulasnella calospora–Serapias vomeracea symbiosis provides clues about nitrogen pathways in orchid mycorrhizas. New Phytol 213, 365–379) and possibly trehalose (Li et al., 2022. Genomes of leafy and leafless Platanthera orchids illuminate the evolution of mycoheterotrophy. Nat Plants 8, 373–388).

 On question 5, this is 28 000 orchids estimated by now and a more specific, accurate ref here would be Selosse et al., 2022 (The Waiting Room Hypothesis revisited: Were orchid mycorrhizal fungi recruited among root endophytes? Annals of Botany 129: 259–270). For the heterotrophic development (you may introduce here the word mycoheterotrophic), a review by Dearnaley et al., 2016. Structure and development of orchid mycorrhizas, *in* F. Martin (ed.) Molecular mycorrhizal symbiosis, p. 63-86. Wiley-Blackwell, Hoboken, New Jersey. ‘These are clear examples that C transfer from CMN to plants does occur’: no, it is most of the time not sure that the C extracted from fungi by protocorm is gained from surrounding plants (rhizoctonias have saprobic capacities). Yet, this is the case in mycoheterotrophic and mixotrophic seedlings (orchids and other), which are linked to ecto- or arbuscular- mycorrhizal fungi - in which case C transfer also occurs in adult plants (V. Merckx (ed.) 2013. Mycoheterotrophy: the biology of plants living on fungi, p. 297-342 (chap. 8). Springer, Berlin Heidelberg). I would avoid the issue of germination in orchids (which are not the sole to display mycoheterotrophic germination, by the way) but I would detail more mycoheterotrophic and mixotrophic (forest) plants, see my comments on this issue below*. Finally, on question 5, we already, with S. Simard and others, pointed the fungus-centered viewpoint that is often overlooked in a 2005 review (Mycorrhizal networks: les liaisons dangereuses. *Trends in Ecology and Evolution* **11** : 621-628).

 *Finally, even if these are not trees, I would mention mycoheterotrophic and mixotrophic (= partially mycoheterotrophic) plants as they provide definite evidence of C transfer. By the way, the Limodorum we analyzed with M. Girlanda is not mixotrophic, but demonstrated partly photosynthetic – so, mixotrophic. Mycoheterotrophy and mixotrophy are currently too fast and superficially reported. One main problem (eg. in question 32) is the net contribution of C transfer to the receivers’ C budget. In mycoheterotrophs, this is 100%. In mixotrophs, this can be estimated from 13C content when the network is ectomycorrhizal, and rages from 0 to nearly 100% (see review in Selosse & Roy, 2009. Green plants eating fungi: facts and questions about mixotrophy. Shading has impact by increasing fungal exploitation (Matsuda et al., 2012. Seasonal and environmental changes of mycorrhizal associations and heterotrophy levels in mixotrophic Pyrola japonica (Ericaceae) growing under different light environments. American Journal of Botany 99: 1177-1188). *Trends in Plant Sciences* **14**: 64-70); more over some achlorophyllous variant sometime survive in natura for mixotrophic species, in which 100% of C is of fungal origin (see Selosse & Roy, 2009, and Lallemand et al., 2019 already cited above).

Where applicable, are recommendations and next steps explained clearly for others to follow? (Please consider whether others in the research community would be able to implement guidelines or recommendations and/or constructively engage in the debate)

Not applicable

Does the article adequately reference differing views and opinions?

Partly

Are all factual statements correct, and are statements and arguments made adequately supported by citations?

Yes

Is the rationale for the Open Letter provided in sufficient detail? (Please consider whether existing challenges in the field are outlined clearly and whether the purpose of the letter is explained)

Yes

Is the Open Letter written in accessible language? (Please consider whether all subject-specific terms, concepts and abbreviations are explained)

Yes

Reviewer Expertise:

Symbiosis, mycology, mycorrhizas and their networks

I confirm that I have read this submission and believe that I have an appropriate level of expertise to confirm that it is of an acceptable scientific standard, however I have significant reservations, as outlined above.

## References

[1] : Mycorrhizal networks: common goods of plants shared under unequal terms of trade. *Plant Physiol* .2012;159(2) : 10.1104/pp.112.195727 789-97 10.1104/pp.112.195727 22517410 PMC3375941

[2] : In situ transcriptomic and metabolomic study of the loss of photosynthesis in the leaves of mixotrophic plants exploiting fungi. *Plant J* .2019;98(5) : 10.1111/tpj.14276 826-841 10.1111/tpj.14276 30735596

[3] : Fungal and plant gene expression in the Tulasnella calospora-Serapias vomeracea symbiosis provides clues about nitrogen pathways in orchid mycorrhizas. *New Phytol* .2017;213(1) : 10.1111/nph.14279 365-379 10.1111/nph.14279 27859287

[4] : Genomes of leafy and leafless Platanthera orchids illuminate the evolution of mycoheterotrophy. *Nat Plants* .2022;8(4) : 10.1038/s41477-022-01127-9 373-388 10.1038/s41477-022-01127-9 35449401 PMC9023349

[5] : Structure and development of orchid mycorrhizas.2016; 10.1002/9781118951446.ch5 63-86 10.1002/9781118951446.ch5

[6] : Mycorrhizal networks: des liaisons dangereuses?. *Trends Ecol Evol* .2006;21(11) : 10.1016/j.tree.2006.07.003 621-8 10.1016/j.tree.2006.07.003 16843567

[7] : Seasonal and environmental changes of mycorrhizal associations and heterotrophy levels in mixotrophic Pyrola japonica (Ericaceae) growing under different light environments. *Am J Bot* .2012;99(7) : 10.3732/ajb.1100546 1177-88 10.3732/ajb.1100546 22739710

# Reviewer report: Reviewer response for version 1

**DOI:** 10.21956/openreseurope.17913.r35973

**Published:** 2023-12-22

**Authors:** Jason D. Hoeksema

**Affiliations:** 1 Department of Biology, University of Mississippi, University Park, Mississippi, USA

We welcome the contribution of this paper to the discussion about how to interpret results of experiments on common mycorrhizal networks (CMNs). However, we disagree that ‘factual evidence about belowground C transfer across CMNs is solid and accumulating.’ There is currently too much uncertainty in interpretation of approved research to make such statements. The authors themselves imply this in the answers they provide to their five questions. In those answers the authors describe many factors that make interpretation of earlier results complicated. As just one example, when discussing Question 1, the authors conclude:

*'The ecological significance of C transfer among different tree species can be masked by the complexity of host tree roots and CMNs.'*

 We agree that the ecological significance of C transfer of CMNs is difficult to determine; however, that does not mean that we can assume that the ecological significance is high. It could just as likely be low. We simply do not know.

 Below, we raise additional questions about interpretations of past experiments and highlight how they are not conclusive evidence for the transfer of carbon (C) through CMNs. We agree with the authors that further research is needed in this area. Indeed, the conclusion of the Karst et al. (2023) review was that far more research is needed on the function of CMNs in forests, given the many confounding factors in field experiments on CMNs. Therefore, we recommend that the title of the current paper be revised to “Belowground carbon transfer across mycorrhizal networks among trees: A possibility worthy of further investigation”

**Abstract:**

*‘Carbon (C) transfer across a common mycorrhizal network (CMN) was demonstrated … later in the field (Simard et al. 1997).’*

 We agree that C moved belowground in the experiment by Simard et al. (1997), but whether it was through a CMN is inconclusive for the reasons explained in Karst et al. (2023) and Henriksson et al. (2023).

*‘…including evidence for ecological significance of carbon transfer (Klein et al. 2016).’*

 First, the Klein et al. (2016) study attempted to estimate the quantity of C transferred through CMNs, but did not attempt to measure the consequences of such C transfer for the growth, survival, or other metric of plant fitness or performance, which would be important for demonstrating the "ecological significance" of carbon transfer.

 Moreover, there are a number of unresolved issues in Klein et al. (2016) precluding conclusive interpretations on the amounts and the belowground pathways of C movement:

 a) Would the high concentration of CO _2_ (mean 530-590 ppm) applied to the labelled trees inflate the amount of C transferred belowground? As Henriksson et al. (2023) point out, it is possible that the donor trees had enhanced capacity to fix C and export photosynthates to their mycorrhizal mycelium, and hence potentially to other trees.

 b) Did the ‘controls’ actually account for the soil pathway of C transfer? Klein et al. (2016) rely on two results to conclude that there was no C moving via the soil pathway (e.g., turnover of litter by soil biota and the subsequent redistribution of its C, and transport in soil water by diffusion and mass flow). First, they compared δ ^13^C  in saprotrophic versus mycorrhizal sporocarps and reported zero δ ^13^C labeling in saprotrophic fungi and decreasing mycorrhizal δ ^13^C  with decreasing distance from the labeled *Picea*. However, the isotopic signatures of  sporocarps and roots cannot be compared because the sporocarps had been sampled four years prior to the roots. Specifically, sporocarps were sampled one to two years after labelling was initiated (2010 & 2011) (Mildner et al. 2014) and fine roots were sampled five and six years after labelling commenced (2015) (Klein et al. 2016). Had the sporocarps been sampled in 2015 along with the fine roots, it is quite likely that some label would have shown up in the saprotrophic species. The lack of change in δ ^13^C  in saprotrophs at increasing distances from labelled trees (averaged across distances in Klein et al. (2016), Fig. 1) may be an artefact of sampling time and not necessarily an indication that C does not move through the soil pathway. Furthermore, the sporocarps were sampled from different locations than the roots (i.e., from different distances around a single labelled tree, not from around labelled and unlabelled trees).

 Second, they sampled δ ^13^C in rhizomes of arbuscular mycorrhizal (AM) understory plants, compared it to the fine roots of labelled/adjacent roots of ectomycorrhizal (EM) canopy trees, and reported that ‘there was absolutely no signal difference between samples collected around unlabelled and labelled *Picea*, and no difference between years.’ Would we expect underground stems (rhizomes) to take up C compounds from the soil in the same amounts as would fine roots of canopy trees? The rhizomes of the understory plants were peeled to remove the bark before analysis, and this would have removed any fine roots present.

 One other issue to consider that we and Henriksson et al. (2023) point out in our reviews, is that the higher label intensity in EM plants could be due to more effective scavenging of labeled compounds because of greater hyphal density compared with the AM plants. So, is the absence of label in rhizomes of an AM understory plant a robust control for the soil pathway?

 ‘ *Diffusional mass flow in unsaturated soil is in the magnitude of m month-1, and its temporal dynamics rarely match those of the observed label transfer (Avital et al., 2022).'*

 Diffusion and mass flow are two separate mechanisms for solute movement in soil, but their rates differ substantially. The authors should clarify which they are referring to, although mass flow is likely more important, given that rates can be much faster than diffusion. Diffusion rate will vary depending on the type of solute. A rate of m per month ^-1^ could explain a substantial proportion of long-distance movement of organic molecules in the field.

 It is notable that Avital et al. (2022) acknowledge, when referring to diffusion, that “ *Several cases of carbon* *transfer demonstrated in our results certainly fit these dynamics, …”*, while concluding that other results were better explained by a faster mechanism, such as transport through CMNs. The authors of the current paper do not acknowledge this caveat.

*‘…CMN-C transfer from mature trees to seedlings has been demonstrated (Orrego 2018)’.*

 To summarize Oreggo (2018), an MSc thesis from the University of British Columbia, eight 'mother' hemlock trees were selected, and a fallen log within 10 m was identified around each tree. Naturally regenerated hemlock seedlings were growing on the logs. A portion of each log, i.e., a ‘control’, was cut and placed on some boards (to severe CMN connections from ‘mother’ trees). ^13^C-labelled glucose was injected into the phloem of each ‘mother’ tree. On days 9 and 15, seedlings on intact and raised logs and the forest floor were analyzed for ^13^C enrichment. Seedlings on intact logs and the forest floor were enriched. There was no significant enrichment of ^13^C for seedlings growing on control logs. In the thesis and in the book 'Finding the Mother Tree’ (Simard 2021), these results are interpreted as ‘mother’ trees providing carbon to their seedlings through CMNs. However, cutting logs and raising them onto boards would not only disconnect CMNs between seedlings and adjacent trees, it would also disrupt the soil pathway. It is not possible to conclude that CMNs mediate C transfer with this experimental set-up, as there was no control for the soil pathway.

*‘…have suggested that trees in forests are more connected than once thought (Simard 2021).’*

 This book is a non-peer-reviewed memoir; therefore, it is not an appropriate source to be cited for scientific evidence in this paper. And as Robinson et al. (2023) point out, there are several misleading claims regarding CMNs in the book.

**Plain language summary:**

*‘Recently, Cahanovitc et al. (2022) showed unequivocally, using DNA-stable isotope probing, 13C in the DNA of specific mycorrhizal species colonizing roots of both donor and recipient saplings, growing in forest soil under natural conditions.’*

 In Cahanovitc et al. (2022), there is no treatment to control for C transferred through root exudates. The ‘control’ treatment prevents the formation of both CMNs and C transfer through soil solution. The ‘treatment’ allows for CMN formation and C transfer through soil solution. Unfortunately, the mechanisms are confounded in this set-up. Passive diffusion may be slow but the distance between hyphae extending from mycorrhizal root tips is likely to be very small. That is, it is not the distance between root tips that is relevant, it is the distance between the tips of hyphae colonizing roots that matters. Other experiments testing the effects of CMNs on C transfer have shown that CMNs are not essential for belowground C transfer to occur (e.g., Simard et al. 1997a, Teste et al. 2010, Pickles et al. 2016). That is, even in the absence of CMNs, C moves belowground, providing evidence for the soil pathway.  

*‘Simard et al. (1997a) also found a fraction of C label transferred to tree seedlings not involved in a CMN compared to those that were.’*

 In the abstract of Simard et al. (1997a) it states ‘Neither net nor bidirectional transfer differed between severing treatments, leaving in question the relative importance of EM hyphae versus soil transfer pathways.’ Thus, this study is not conclusive evidence for C moving through CMNs. Perhaps the authors meant to refer to Simard et al. 1997b?

*‘Hence, the significance of the CMN-C transfer is probably more nuanced, e.g., in providing C for osmoregulation (Sapes et al., 2021) or defense metabolite transfer (Song et al., 2015).’*

 In Sapes et al. (2021), the CMN treatments didn’t work as planned, so there was no comparison between seedlings with and without CMNs. In Song et al. (2015), a pot study, there is evidence of C transfer through CMNs, and for subsequent upregulation of potential defensive enzymes, but both of those effects are negated when roots are allowed to intermingle, as they would in a real forest. This is a fascinating result, but is the only experiment testing this mechanism in trees, and does not support stating that CMN-mediated C transfer would have ecological significance in a real forest.

*‘Specific mycorrhizal fungi transfer C among trees, including from canopy trees to seedlings, or from sunlit saplings to shaded saplings of the same, or different (non-kin), species. This facilitative behavior has been demonstrated by Teste et al. (2009) and Bingham & Simard (2012) in temperate forests...’*

 The idea of kin vs non-kin (i.e., whether donor and recipient trees/saplings/seedlings are genetically related) is being confused here with whether the donor and recipient trees/saplings/seedlings were of the same or different species. The meaning should be clarified.

 Neither of the studies cited used a shading treatment, and neither considered the genetic relatedness of the donors and receivers. Both were performed in logged sites, so all seedlings were sunlit. Most importantly, neither of these studies investigated C transfer, only effects of CMNs on seedling survival and other characteristics.

 Bingham and Simard (2012) found that ‘Seedling survival decreased when seedlings were unable to form a network’. However, alternative interpretations are (i) that the soil volume available to foraging hyphae was smallest in the 0.5 µm mesh treatment, thereby reducing seedling survival in the control treatment, or (ii) that the mesh treatments altered the composition or diversity of the EM fungal community (as found by Teste et al. 2009a & 2009b) or pathogenic fungal communities in ways that affected seedling survival.

*‘C transfer takes place between different, unrelated tree species sharing mycorrhizal species (Avital et al., 2022; Rog et al., 2020).’*

 Rog et al. (2020) provides good support for C transfer from trees to EM fungi. However, we question that the magnitude of belowground C transfer was correlated with EM fungal community similarity, as the analyses on which this claim was based eliminated noise by averaging within species and by dropping extreme values from what was already a small data set. We also note that in Avital et al. (2022), there was no significant correlation between the δ ^13^C level in the recipients and the abundance of mycorrhiza fungal ASVs shared with the donor tree. Altogether, it doesn’t appear that the relationship between EM fungal community similarity and C transfer is clear across these two studies.

*‘This includes C transfer between an EM-host to an AM-host with dual mycorrhization status (Cupressus sempervirens; Avital et al., 2022).’*

 Was it confirmed that *Cupressus sempervirens* was colonized by both ecto- and arbuscular mycorrhizal fungi in the experiment? Without these data, we agree with Henriksson et al. (2023): that C transfer in this study most likely occurred via non-mycorrhizal pathways.

**Other issues:**

*'Thus, a fungus→plant C movement is not unlikely when C is tied to nutrients (e.g., amino acids) or when trees or plants acquire C from degenerating hyphae in the Hartig Net (as in AMF arbuscules).*'

 References should be provided for this statement. We are not away of studies that have shown that the Hartig net degenerates and provides C to EM plants.

*'However, there is sufficient evidence that trees in forests are connected by a CMN and transmitting C among themselves, …'*

 We agree that **it is likely** that many trees in forests are connected by CMNs, but we disagree that there is currently sufficient evidence for this statement. Detailed mapping of fungal genets and their mycorrhizal roots using genotyping tools has been conducted in only two tree species, and the temporal dynamics and persistence of CMNs has not been explored, so much more research is required.

 We agree that there is sufficient evidence that C moves belowground between trees. The current sentence structure does not imply that the C is moving through the CMNs, which is appropriate; however, this could be missed during a quick read of the sentence. We recommend that the sentence be re-written so that readers are not confused on the meaning here. Yes, C can move belowground, and, yes, it is likely that some roots of trees are physically connected by a mycorrhizal fungal mycelium; however, linking those two in a functional way is premature.

Where applicable, are recommendations and next steps explained clearly for others to follow? (Please consider whether others in the research community would be able to implement guidelines or recommendations and/or constructively engage in the debate)

Not applicable

Does the article adequately reference differing views and opinions?

Yes

Are all factual statements correct, and are statements and arguments made adequately supported by citations?

Partly

Is the rationale for the Open Letter provided in sufficient detail? (Please consider whether existing challenges in the field are outlined clearly and whether the purpose of the letter is explained)

Yes

Is the Open Letter written in accessible language? (Please consider whether all subject-specific terms, concepts and abbreviations are explained)

Yes

Reviewer Expertise:

Ecology and evolution of mycorrhizal symbiosis; experimental approaches to studying common mycorrhizal networks

I confirm that I have read this submission and believe that I have an appropriate level of expertise to confirm that it is of an acceptable scientific standard, however I have significant reservations, as outlined above.

## References

[1] : Asymmetric belowground carbon transfer in a diverse tree community. *Mol Ecol* .2022;31(12) : 10.1111/mec.16477 3481-3495 10.1111/mec.16477 35451146 PMC9325067

[2] : Ectomycorrhizal Networks of Pseudotsuga menziesii var. glauca Trees Facilitate Establishment of Conspecific Seedlings Under Drought. *Ecosystems* .2012;15(2) : 10.1007/s10021-011-9502-2 188-199 10.1007/s10021-011-9502-2

[3] : Ectomycorrhizal fungi mediate belowground carbon transfer between pines and oaks. *ISME J* .2022;16(5) : 10.1038/s41396-022-01193-z 1420-1429 10.1038/s41396-022-01193-z 35042973 PMC9039061

[4] : Re-examining the evidence for the mother tree hypothesis - resource sharing among trees via ectomycorrhizal networks. *New Phytol* .2023;239(1) : 10.1111/nph.18935 19-28 10.1111/nph.18935 37149889

[5] : Positive citation bias and overinterpreted results lead to misinformation on common mycorrhizal networks in forests. *Nat Ecol Evol* .2023;7(4) : 10.1038/s41559-023-01986-1 501-511 10.1038/s41559-023-01986-1 36782032

[6] : Belowground carbon trade among tall trees in a temperate forest. *Science* .2016;352(6283) : 10.1126/science.aad6188 342-4 10.1126/science.aad6188 27081070

[7] : Long-term ¹³C labeling provides evidence for temporal and spatial carbon allocation patterns in mature Picea abies. *Oecologia* .2014;175(3) : 10.1007/s00442-014-2935-5 747-62 10.1007/s00442-014-2935-5 24696359

[8] : Transfer of 13 C between paired Douglas-fir seedlings reveals plant kinship effects and uptake of exudates by ectomycorrhizas. *New Phytol* .2017;214(1) : 10.1111/nph.14325 400-411 10.1111/nph.14325 27870059

[9] : Mother trees, altruistic fungi, and the perils of plant personification. *Trends Plant Sci* .2023; 10.1016/j.tplants.2023.08.010 10.1016/j.tplants.2023.08.010 37735061

[10] : Share the wealth: Trees with greater ectomycorrhizal species overlap share more carbon. *Mol Ecol* .2020;29(13) : 10.1111/mec.15351 2321-2333 10.1111/mec.15351 31923325 PMC7116085

[11] : Plant carbohydrate depletion impairs water relations and spreads via ectomycorrhizal networks. *New Phytol* .2021;229(6) : 10.1111/nph.17134 3172-3183 10.1111/nph.17134 33280134

[12] : Reciprocal transfer of carbon isotopes between ectomycorrhizal Betula papyrifera and Pseudotsuga menziesii. *New Phytol* .1997;137(3) : 10.1046/j.1469-8137.1997.00834.x 529-542 10.1046/j.1469-8137.1997.00834.x 33863069

[13] : Net transfer of carbon between ectomycorrhizal tree species in the field. *Nature* .1997;388(6642) : 10.1038/41557 579-582 10.1038/41557

[14] : Defoliation of interior Douglas-fir elicits carbon transfer and stress signalling to ponderosa pine neighbors through ectomycorrhizal networks. *Sci Rep* .2015;5: 10.1038/srep08495 8495 10.1038/srep08495 25683155 PMC4329569

[15] : Access to mycorrhizal networks and roots of trees: importance for seedling survival and resource transfer. *Ecology* .2009;90(10) : 10.1890/08-1884.1 2808-22 10.1890/08-1884.1 19886489

[16] : Role of mycorrhizal networks and tree proximity in ectomycorrhizal colonization of planted seedlings. *Fungal Ecology* .2009;2(1) : 10.1016/j.funeco.2008.11.003 21-30 10.1016/j.funeco.2008.11.003

[17] : Net carbon transfer betweenPseudotsuga menziesii var.glauca seedlings in the field is influenced by soil disturbance. *Journal of Ecology* .2010;98(2) : 10.1111/j.1365-2745.2009.01624.x 429-439 10.1111/j.1365-2745.2009.01624.x

# Reviewer report: Reviewer response for version 1

**DOI:** 10.21956/openreseurope.17913.r35510

**Published:** 2023-10-12

**Authors:** David Perry

**Affiliations:** 1 College of Forestry, Oregon State University, Corvallis, Oregon, USA

The article is an important contribution to the debate on C movement in common mycorrhizal networks (CMN). Recent publications (e.g. Karst *et al.*, cited by the authors of this paper) have raised significant questions about that phenomenon. Most points raised by Karst and others are correct, but unfortunately they have also effectively thrown the baby out with the bathwater, denying what has been demonstrated in numerous published papers. It is important to set the record straight on what is known and what is not, and Klein *et al.* do a good job of that. The question of resource transfer through CMNs may or may not have significant implications for forest growth and health. I think it does, but to move research ahead efficiently we need to acknowledge the potential that has been shown, and we now need to know why it manifests sometimes/places and not others. I wholeheartedly endorse the Klein *et al.* manuscript.

Specific comments:

 Point 5. “ t *ransfer in the form of amino acids ( Simard et al., 2012) inescapably involves N transfer, going both ways. Thus, a fungus→plant C movement is not unlikely when C is tied to nutrients (e.g. amino acids) or when trees or plants acquire C from degenerating hyphae in the Hartig Net (as in AMF arbuscules).”* A couple of points need clarification: 1) *"going both ways”* - That seems to imply the plant can transfer amino acids to the fungus. Is that what you intend? If so, provide a citation. (2) *“(as in AMF arbuscules)” - *I believe what you intend to say is “plants acquire C from degenerating hyphae in the Hartig Net or in AMF arbuscules”.

 “ *Finally, an evolutionary advantage should exist for fungi to maintain diversity of tree hosts and hence C sources ( Tedersoo et al., 2020)*” I’d note this argument was made by Perry (1998 ^1^).

Where applicable, are recommendations and next steps explained clearly for others to follow? (Please consider whether others in the research community would be able to implement guidelines or recommendations and/or constructively engage in the debate)

Yes

Does the article adequately reference differing views and opinions?

Yes

Are all factual statements correct, and are statements and arguments made adequately supported by citations?

Yes

Is the rationale for the Open Letter provided in sufficient detail? (Please consider whether existing challenges in the field are outlined clearly and whether the purpose of the letter is explained)

Yes

Is the Open Letter written in accessible language? (Please consider whether all subject-specific terms, concepts and abbreviations are explained)

Yes

Reviewer Expertise:

Ecological processes. Ecology of mycorrhizal fungi

I confirm that I have read this submission and believe that I have an appropriate level of expertise to confirm that it is of an acceptable scientific standard.

## References

[1] : A moveable feast: the evolution of resource sharing in plant-fungus communities. *Trends Ecol Evol* .1998;13(11) : 10.1016/s0169-5347(98)01456-6 432-4 10.1016/s0169-5347(98)01456-6 21238382

# Response

**Published:** 2023-10-13

**Authors:** tamir klein

**Affiliations:** Plant and Environmental Sciences, Weizmann Institute of Science, Rehovot, Center District, Israel

Dear Prof. David Perry (Reviewer 1), Thank you for your positive assessment of our letter. We appreciate the comments made and will adjust the letter accordingly, once the other additional review reports come in. Sincerely, Tamir Klein
